# RENAL TRANSPLANT IN A CHILD WITH BILATERAL WILMS’ TUMOR NATIONAL PREMIERE

**Published:** 2008-02-25

**Authors:** Sinescu Ioanel, Hârza Mihai, Şerbănescu Bogdan, Gîngu Constantin, Ştefan Bogdan, Dudu Cătălin

**Affiliations:** * Center of Urological Surgery, Dialysis and Renal Transplantation, “Fundeni” Clinical Institute, Bucharest, Romania

**Keywords:** bilateral Wilms’ tumor, children, combined treatment, kidney transplantation

## Abstract

Introduction: Bilateral Wilms’ tumors with an unfavorable histology require a combined treatment (extensive surgery, polychimiotherapy, radiotherapy).

Objective: Presentation of the first renal transplant performed in Romania in a child with bilateral Wilms’ tumor, at 3 years and 4 months after the end of a multimodal treatment.

Material and methods: Patient C. N., born on 30.04.1998, was diagnosed in 04.2001 with right parenchymal renal tumor, polycystic kidney, left cystic renal tumor. 25.04.2001 - right radical nephrectomy and partial left upper pole nephrectomy; histopathology examination: triphasic bilateral nephroblastoma, reactive lymph nodes, negative resection edges in the left kidney. 30.04-19.11.2001- polychemotherapy according to the NWTS-5 stages 2-4 focal anaplasia and radiotherapy of the right kidney bed (29.06.2001). 02.2002- a nephrotic syndrome on the remnant kidney which requires its excision and peritoneal dialysis. Abdominal control CT was normal in 03.2005. 11.03.2005- renal transplant from living related donor.

Results: Favorable post-transplant course with normal renal clearance values; at 2 months, normal urography control.

Conclusions: The tumor pathology does not represent an absolute contraindication for renal transplantation. For the cases with extensive surgery, polychimiotherapy and radiotherapy correctly applied, a pre-transplant “tumor-free” period of at least 2 years is compulsory.

## Introduction

The nephroblastoma [Wilms’ tumor] is the most frequent solid tumor in childhood, representing 5% of all cancers of the child. Concomitant bilateral Wilms’ tumors occur in 5% of cases. Histologically, they are characterized by tissue and cell polymorphism, with various rates of “nephrogenic” (blastemal), epithelial and stromal structures. 

The most important negative prognostic factors remain the unfavorable histological subtypes (clear cell sarcoma, rhabdoid and anaplastic tumors). Bilateral Wilms’ tumors (stage 5) with an unfavorable histology require a combined treatment (extensive surgery, polychemotherapy, radiotherapy). 

Approximately 15% of children with Wilms’ tumors have recognized congenital abnormalities: aniridia, Beckwith-Wiedermann syndrome, genitourinary abnormalities (renal hypoplasia, renal fusion, cystic renal abnormalities, ectopic kidney, hypospadias and cryptorchidism).

Up to 15% of patients with Wilms tumors and congenital malformations with favorable post-therapeutical outcome, will develop a second malignancy due to radiation therapy and/or inherited susceptibility.

## Objective

The article’s objective is the presentation of the first renal transplant performed in Romania in a child with bilateral Wilms tumor, at 3 years and 4 months after the end of a multimodal treatment.

## Material and methods

Male patient, C. N., born on the 30th of April 1998, was investigated in April 2001 for fever (38.5°C) and for the occurrence of an abdominal tumor, the diagnosis (established by ultrasonography, C.T., and renal scintigram) being right parenchymatous renal tumor, polycystic kidney, left cystic renal tumor. 

**Figure 1:**
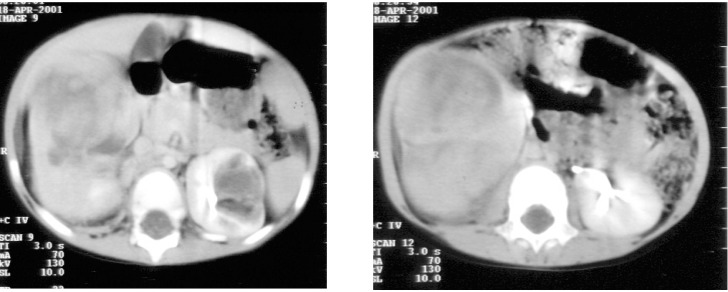
April 2001 - polycystic kidneys, right parenchimatous renal tumor, left cystic renal tumor.

On the 25th of April 2001, a right radical nephrectomy and partial left upper pole nephrectomy were performed; results of the histopathology examination: triphasic bilateral nephroblastoma, reactive lymph nodes, without any tumor invasion at the level of resection edges in the left kidney. 

During the period 30.04-19.11.2001, the patient underwent a PCT treatment according to the NWTS-5 stages 2-4 focal anaplasia, as well as radiotherapy at the level of right kidney bed (29.06.2001). The treatment was additionally burdened by the occurrence of arterial hypertension requiring treatment with Captopril and by a moderate elevation of proteinuria values. 

Abdominal and thoracic CT after 2 months was normal. 

**Figure 2:**
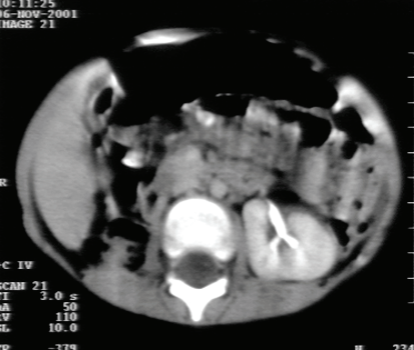
January 2002 - normal abdominal and thoracic CT scans, 2 months after polychemotherapy and radiotherapy.

In February 2002, a nephrotic syndrome develops on the remnant kidney, which does not respond to immunosuppressive treatment and which, associated with uncontrollable arterial hypertension and proteinuria, requires excision of the remnant kidney. 

A hemodialysis program is attempted, but it is interrupted because of arterial hypotension. Patient enters a peritoneal dialysis program, the course of which is complicated by multiple and recurrent abdominal hernias, as well as by two peritonitis episodes (April 2003 and June 2004). 

Abdominal control CT was normal in March 2005. On the 11th of March 2005, kidney transplantation from living related donor (mother) is performed.

**Figure 3:**
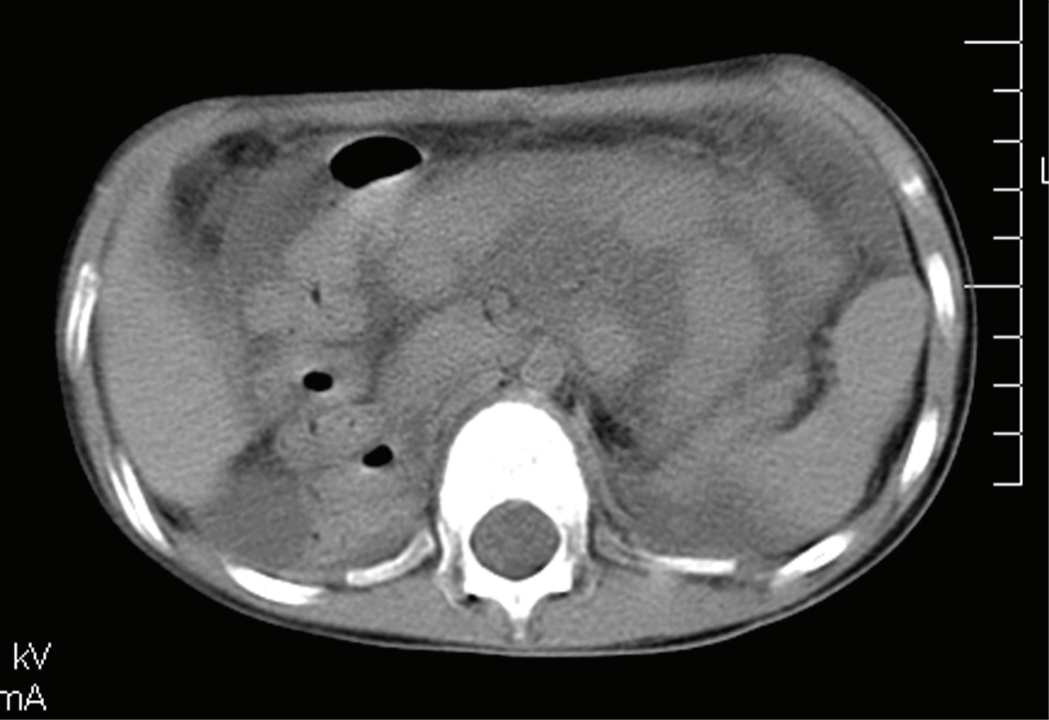
March 2005 - normal cranial, thoracic and abdominal CT scan.

## Results

Favorable post-transplant course without surgical or immunological complications, with normal renal clearance values; at 2 months, normal urographic control; at 12 months, abdominal CT control is normal. Three years after renal transplantation, serum creatinine level is normal, the renal graft is morphologically normal and the child is living with normal parameters according to age.

**Figure 4:**
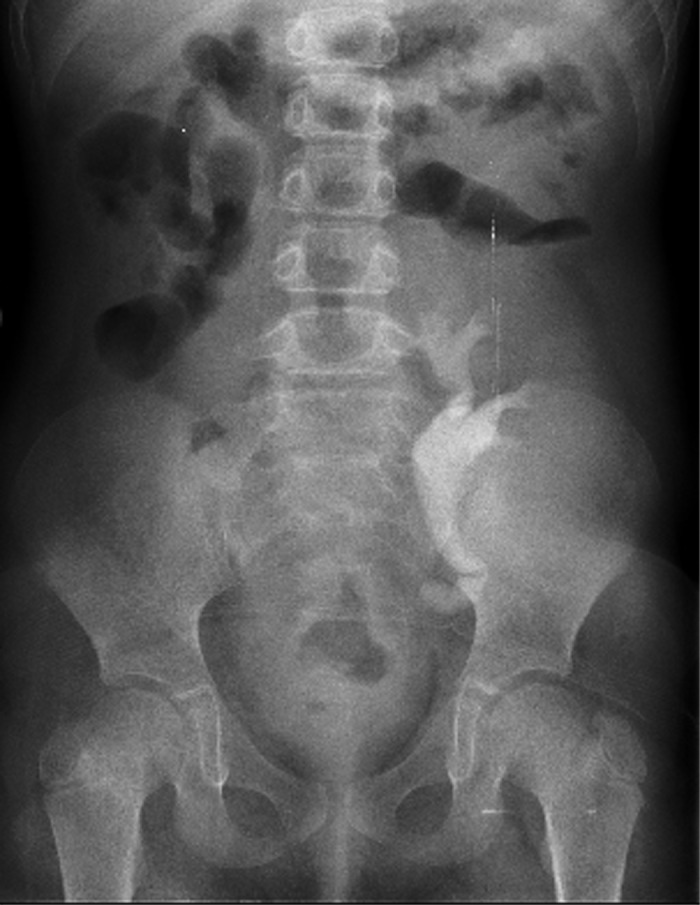
Normal IVP - 2 months after renal transplantation.

## Discussions

The most complete therapeutical option for patients with end stage disease is kidney transplantation.

The tumoral pathology does not represent an absolute contraindication for the renal transplant. 

There are though two major reasons for excluding patients with malignant disease: the first is that immunosuppressive drugs may unfavorably influence the natural history of the malignancy; the second is that it is not reasonable for someone whose life expectancy is significantly curtailed by the presence of malignant disease to undergo transplantation.

But, the multimodal approach of the treatment of children with nephroblastoma has significantly improved outcomes. The IIIrd National Wilms’ Tumor Study (NTWS) reported an overall survival rate without tumoral recurrence of 85-92, 4% and for patients with bilateral Wilms’ tumor revealed a 3-year survival rate of 82%.

For the cases with extensive surgery, polychemotherapy and radiotherapy correctly treated patients with bilateral Wilms’ tumors, a pre-transplant “tumor-free” period of at least 2 years is compulsory.

Also, long-term follow-up is mandatory for patients with pretransplantation nephroblastoma (because of immunosuppressive drugs), especially for children with Wilms’ tumors and associated congenital abnormalities.
